# Experimental Methods to Simulate and Evaluate Postsurgical Peripheral Nerve Scarring

**DOI:** 10.3390/jcm10081613

**Published:** 2021-04-10

**Authors:** Alessandro Crosio, Giulia Ronchi, Benedetta Elena Fornasari, Simonetta Odella, Stefania Raimondo, Pierluigi Tos

**Affiliations:** 1 UO Microchirurgia e Chirurgia della Mano, Ospedale Gaetano Pini, Piazza Andrea Ferrari 1, 20122 Milano, Italy; Alessandro.Crosio@asst-pini-cto.it (A.C.); simonettaodella@gmail.com (S.O.); Pierluigi.Tos@asst-pini-cto.it (P.T.); 2 Department of Clinical and Biological Sciences, Neuroscience Institute of the “Cavalieri Ottolenghi” Foundation (NICO), University of Turin, Regione Gonzole 10, 10043 Orbassano, Italy; giulia.ronchi@unito.it (G.R.); benedettaelena.fornasari@unito.it (B.E.F.)

**Keywords:** scar tissue, peripheral nerve regeneration, antiadhesion devices, animal models

## Abstract

As a consequence of trauma or surgical interventions on peripheral nerves, scar tissue can form, interfering with the capacity of the nerve to regenerate properly. Scar tissue may also lead to traction neuropathies, with functional dysfunction and pain for the patient. The search for effective antiadhesion products to prevent scar tissue formation has, therefore, become an important clinical challenge. In this review, we perform extensive research on the PubMed database, retrieving experimental papers on the prevention of peripheral nerve scarring. Different parameters have been considered and discussed, including the animal and nerve models used and the experimental methods employed to simulate and evaluate scar formation. An overview of the different types of antiadhesion devices and strategies investigated in experimental models is also provided. To successfully evaluate the efficacy of new antiscarring agents, it is necessary to have reliable animal models mimicking the complications of peripheral nerve scarring and also standard and quantitative parameters to evaluate perineural scars. So far, there are no standardized methods used in experimental research, and it is, therefore, difficult to compare the results of the different antiadhesion devices.

## 1. Introduction

Scar tissue around the nerve can arise as a consequence of traumatic injuries and surgical procedures on peripheral nerves. This condition easily worsens the capacity of the peripheral nerve to regenerate and can give rise to traction neuropathies. Nerve tethering in the surgical scar is still the main cause of symptoms related to perineural scarring [1].

Traction neuropathies can be the consequence of elective procedures, including nerve decompression, primary nerve repair, and so on. For instance, 7–20% of patients subjected to primary median nerve release report pain and symptom recurrence [2,3]. Thus, peripheral nerve injuries compromise the quality of life of affected people, with a consequent important socioeconomic impact [4,5,6].

This condition is difficult to manage; according to different reports, compression symptoms persist after 40–90% of revision procedures, and 20% of patients actually require a third operation [7]. Moreover, 5% of nerve sutures have been estimated to induce a pain syndrome [8].

A primary role in this pathological condition has been attributed to the formation of scar tissue around the injured nerve. In particular, extrinsic nerve scarring occurs at the periphery of the epineurium, while intrinsic nerve scarring occurs within the nerve and can surround neural structures at all levels (both perineurium and endoneurium) [9]. To overcome scar tissue formation, a lot of different antiadhesion devices have been tested, developed, and introduced in clinical practice.

Since the late 1990s, researchers have tried to develop experimental models to investigate the efficacy of different antiadhesion devices. Several types of devices have been tested so far, but the methods employed to produce, simulate, and evaluate postsurgical scars are completely inhomogeneous and not reproducible [10]. This makes it difficult to compare the efficacy of the different antiadherence strategies in order to optimize clinical treatment.

The aim of this review is to illustrate the different methods adopted in experimental research to simulate and evaluate postsurgical scars. Finally, an overview of the antiadhesion devices tested so far in preclinical research is also provided.

## 2. Materials and Methods

An extensive research on PubMed was performed, employing the following search string: “peripheral nerves AND fibrosis OR perineural scar OR scar neuropathy OR traction neuropathy AND prevention”, limited to English, other animals, and between 1 January 1995 and 31 December 2020. Furthermore, the reference list of each article was screened in order to find any additional original articles. Selection by title, abstract, and text was then performed; a total of 60 papers were retrieved. We did our best to include all articles available; nevertheless, inadvertently, we could have missed some papers, and we apologize in advance to their authors.

## 3. Scar Simulation: Animal and Nerve Choice

The choice of an appropriate animal model for preclinical research depends on different factors, including the aim and duration of the study, the anatomy and physiology of the animal model, the size of the medical device that needs to be tested, and, of course, the similarity with human clinical characteristics of the disease/condition. Finally, the cost and care of the animal model (housing, feeding, and caring) can also be considered.

For the study of peripheral nerve scarring, the most employed animal model is the rat model, followed by mouse and rabbit used in a limited number of studies (see Tables 1–6). This can be due to the lower cost of rats compared to rabbits, the easier caregiving, and the faster scar formation in smaller rather than bigger animals. On the other hand, mice (and their nerves) are very small and, therefore, more difficult to manage. Additionally, different rat/rabbit/mouse strains have been used. No research with other animal species (sheep, pigs, monkeys, cats, or dogs) has been found, in contrast with studies on peripheral nerve regeneration, where sheep as an animal model is often used to test regeneration across long distances [11,12]. 

Moreover, the choice of nerve model can be guided by several factors, including nerve size and the surrounding tissues. Most researchers use the sciatic nerve because it can be easily dissected, and there are no surrounding vascular and nervous structures that can impair the efficacy of the study. Only a few papers have used other nerve models such as the ulnar [13,14], peroneal [15], and median nerves [16]. 

## 4. Scar simulation: Experimental Methods to Induce Scar Formation

The main aim of most of the research dealing with scar formation is to test the efficacy of antiadhesion devices. Only very few papers [17,18,19,20,21] are focused on the standardization of a scarring method without testing any antiadhesion device (Table 1). These papers are very important in this field because a shared, effective, reliable, and reproducible protocol to induce and evaluate the amount of scar tissue is needed to compare the efficacy of different antiadhesion devices; so far, this is not available [18,20,21,22].

**Figure 1:**
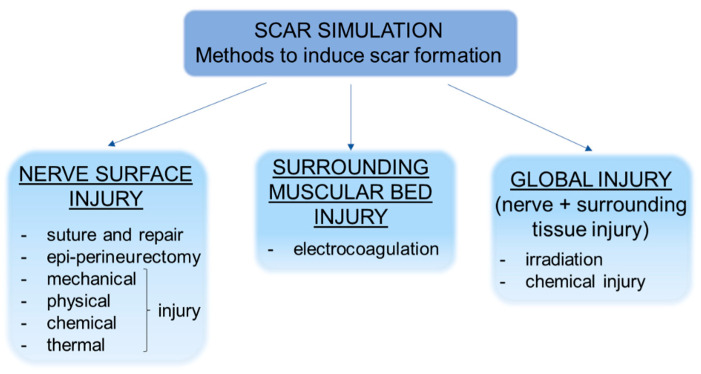
Representative scheme showing the different strategies to simulate scar formation in preclinical models.

The methods used to induce scar formation are several (Figure 1), but two of these are more frequently used. The first one consists of a direct injury (mechanical, epiperineurectomy, suture and repair, thermal, chemical, or physical) applied to the nerve surface. The second one consists of inducing an injury in the surrounding muscular bed by means of electrocoagulation, triggering the process of fibrosis from the surrounding tissue. Some researchers have induced a global injury to the nerve and surrounding tissues by scratching both nerve and muscles with irradiation or chemical injuries. Furthermore, the envelopment of the nerve in a silastic tube in order to let the scar tissue rise has been proposed.

Some of the earlier papers [23,24,25] performed a two-stage procedure (first stage injury, second stage neurolysis and antiadhesion application), which is a more traumatic experience for animals, without evidence of increased efficacy compared to a one-stage procedure. With respect to the 3Rs statement [26], a single-stage experiment can have the same efficacy as a two-stage one.

## 5. Scar Evaluation: Methods to Evaluate Scar Formation in Experimental Models

**Figure 2:**
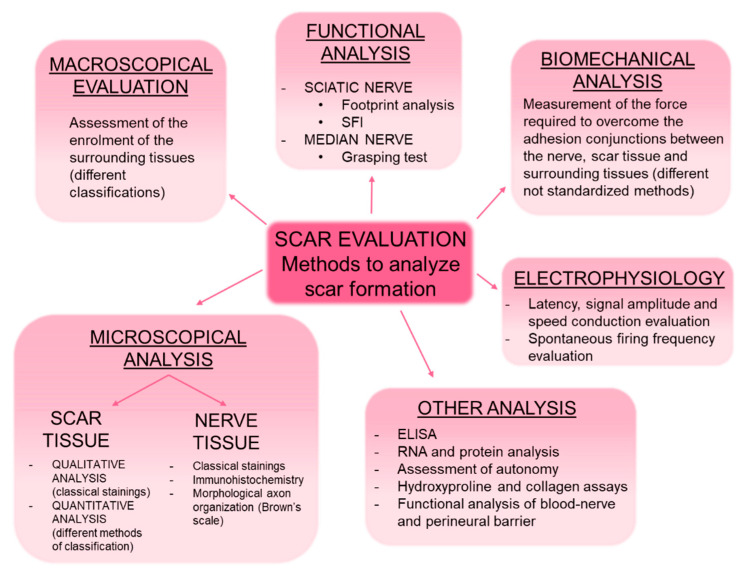
Representative scheme showing the different methods of evaluating scar formation in preclinical models. SFI (sciatic functional index), ELISA (enzyme-linked immunosorbent assay).

Different procedures can be used to evaluate scar formation, including gross examination of the scar tissue, microscopical analysis of the nerve and surrounding tissue, functional tests, and electrophysiological and biomechanical evaluations (Figure 2). All of these evaluation methods are combined differently by authors. It must also be noted that the time points analyzed are very different among the studies, ranging from few days to several months from the induction of scar formation; the research also differs according to the employed animal model.

### 5.1. Macroscopical Analysis

Gross evaluation is the first fundamental step to macroscopically grade scar tissue; it aims to assess the enrolment of the surrounding tissues (including skin, muscles, and deep tissues) in the compression of the nerve and the collaboration with the newborn perineural scar tissue.

Different classifications have been proposed by different authors to evaluate the degree of scar formation. The most used and complete classification is the numeric grade scheme defined by Petersen [27]. This classification allows us to evaluate closure of skin and muscle fascia (Grade 1: skin or muscle fascia entirely closed; Grade 2: skin or muscle fascia partially open; Grade 3: skin or muscle fascia completely open) and to evaluate nerve adherence (Grade 1: no dissection or mild blunt dissection; Grade 2: some vigorous blunt dissection required; Grade 3: sharp dissection required). Another adopted grading scheme is the 4-point qualitative scale that evaluates the perineural adhesions and type of dissection required to achieve complete neurolysis, as follows: absent or thin adhesions—delicate blunt (score 0); mild adhesion—vigorous blunt (score 1); moderate adhesion—delicate sharp (score 2); severe adhesion—difficult sharp (score 3) [13,14,23,28].

Abe [17] proposed a classification of nerve adhesion based on Fontana’s band (an optical manifestation of axonal undulations characteristic of peripheral nerves). They classify nerve adhesion as Group I (nonadhesion group) when the bands appear and Group II if they are not visible. Additionally, Group II is divided into Group IIa when a thickening of the epineurium and perineurium is observed (but not endoneurial fibrosis) and Group IIb when endoneurial fibrosis is observed.

Finally, some authors have reported the presence or absence and qualitative observations of scar tissue around the nerve without grading it.

### 5.2. Microscopical Analysis: Histological Staining and Immunohistochemistry

Microscopical analysis is employed by most authors and consists mainly of the use of different histological stainings to visualize and describe the different structures involved (not only the scar tissue but also the nerve and surrounding tissues, such as muscle), both qualitatively and quantitatively (see Tables 1–6).

#### 5.2.1. Analysis of the Scar Tissue

The most employed method to highlight scar tissue is Masson’s trichrome staining because it specifically marks collagen in green/blue and can be easily distinguished from other structures such as muscle fibers (stained in red), cytoplasm (light red or pink), and cell nuclei (dark brown to black).

Haematoxylin and eosin (H&E) is the most widely used staining for histological purposes because it provides a general overview of the tissue, and it is mainly used to distinguish nerves from surrounding tissues. It is the combination of two histological stains: hematoxylin, which stains cell nuclei in blue/dark-purple, and eosin, which stains cytoplasm in pink, and other structures, including extracellular matrix and collagen, in shades of pink.

Picrosirius red is often used since it selectively highlights collagen fibers [29]; indeed, this dye allows us to visualize collagen fibers in red (specific for collagen types I and III), while the other structures are stained in yellow (nuclei, cytoplasm, muscular fibers, red blood cells). The Gordon and Sweet technique, used to reveal reticulinic acid, a collagenous tissue marker, has also been adopted, as well as chromotrop-aniline-blue, which stains collagen in blue and muscle fibers in red.

Besides a qualitative analysis of scar tissue formation, histological staining allows us to also perform quantitative analyses. One of the most used parameters to measure scar tissue is the scar tissue formation index, calculated by dividing the mean thickness of the scar tissue by the mean thickness of the nerve tissue [18,27,28,30,31,32,33,34,35,36,37,38,39,40,41]. Sakurai’s classification of neural fibrosis [42] allows us to describe the extension of the perineural scar from epineurium to endoneurium (Grade 1: the nerve is normal; Grade 2: extraneural type; Grade 3: intraneural, epineural type; Grade 4: intraneural, perineural type; Grade 5: intraneural, endoneurial type; Grade 6: dispersive type) [17].

The number of fibroblasts/fibrocytes is another parameter that is often used, and it allows us to classify the specimens into Grade 1—less than 100 fibroblasts; Grade 2—100–150 fibroblasts; Grade 3—more than 150 fibroblasts [30,32,34,41,43,44].

The classification of Ornelas [45] has also been adopted [46,47], and it allows us to classify extraneural and intraneural fibrosis; extraneural fibrosis is classified into Grade 1—absent or minimal fibrosis; Grade 2—moderate fibrosis; Grade 3—major fibrosis. Intraneural fibrosis is classified into Grade 1—the presence of fibrous tissue between the nerve fibers; Grade 2—fibrous tissue partially blocking the passage of nerve fibers; Grade 3—fibrous tissue completely interrupting the passage of nerve fibers.

Dam-Hieu [28] calculated the thickness of the dense scar surrounding the nerve. The largest thickness of the scar ring (ST) was measured. This value was then normalized by dividing it by the nerve diameter (ND). The authors call this value the fibrotic index (fibrotic index = 2 ST/ND).

Other quantitative or semiquantitative analyses have also been proposed, such as the average thickness of collagen in the epineurium [14,48], the count of fibroblasts and inflammatory cells [36,38,46], the thickness of the epifascicular epineurium, the amount of connective tissue in the interfascicular and epifascicular epineurium [49], and the percentage of area of staining (PAS) calculation by outlining the intraneural tissue [50,51,52,53].

#### 5.2.2. Analysis of the Nerve Tissue

To investigate the nerve tissue specifically, Luxol fast blue, P-phenylenediamine, silver staining, the Weil method, and toluidine blue were used to describe axon distribution and highlight the myelin sheath. In particular, toluidine blue staining offers the possibility of performing morphoquantitative analyses to estimate the number of myelinated fibers, axon density, fiber and axon diameter, myelin thickness, and g-ratios (axon-diameter/fiber-diameter), which can be correlated with functional recovery [5,54].

Moreover, the longitudinal histomorphological organization of the axon at the nerve repair site can be evaluated according to the scale developed by Brown et al. [55] and adopted by several authors [31,35,36,38,56]: Grade 1—failure, no continuity of the axons from the proximal to the distal ends; Grade 2—poor organization (interlacing or whirling appearance of the nerve fibers); Grade 3—fair organization (focal whirling appearance, focal parallel alignment); Grade 4—good organization, approaching normal (mostly parallel, without a whirling or wavy appearance); Grade 5—excellent organization of the repair site, indistinguishable from the norm.

Finally, some authors have added immunohistochemistry to classical staining methods. This method is efficient in describing nerve regeneration quality with antibodies, which specifically mark Schwann cells (anti-S100, to mark the myelin sheath) [21,36], nerve cones (anti-GAP 43) [37], or antineurofilament [21,57]. Immunohistochemistry has been also adopted to study perineural scars by using antibodies against TGF-β markers of macrophages [58], CD68 for activated macrophages [21,37,57,59], anti-CCR7 for proinflammatory M1 macrophages [57], CD3, CD8 [21], and collagen I [60]. Additionally, antibodies against decorin, aggrecan, laminina 2, collagen IV, and fibronectin have been used [61]. Finally, Murakami [62] performed immunohistochemistry analysis on dorsal root ganglia using the inflammation marker CGRP and tissue stress ATF3 antibodies.

### 5.3. Functional Analysis

Some studies performed functional analyses, even though these analyses are not precise for scar quantification and we are not sure that they can be directly correlated to the amount of scarring observed around the nerve.

Most of the studies dealing with peripheral scarring use the sciatic nerve model, foot print analysis, and the Sciatic Function Index (SFI) as the most adopted tests [63]. Other parameters evaluated are allodynia by means of von Frey filaments [62] and walking patterns induced by pain with the CatWalk system [21,51,62].

The only study that used the median nerve model tested the function of the nerve by means of the grasping test [16].

### 5.4. Electrophysiological Study

Electrophysiology is another test that can be used in order to analyze the formation of scars around the nerve. Indeed, compression around a nerve causes pathophysiological changes that can be registered with this assessment.

The electrophysiological analysis is based on compound motor action potential (CMAP). Different aspects of electrical activity can be registered, such as latency, signal amplitude, and speed conduction. These parameters correlate with nerve conduction; the more the scar is present, the more these parameters are altered.

Another assessment that is useful to evaluate is the frequency of spontaneous firing because it has been demonstrated to be related to nerve suffering: the more frequent the firing is, the more the nerve is suffering [64].

Zuijdendorp [22] and its colleagues performed the evaluation by means of magnetoneurography of first peak amplitude, peak–peak amplitude, area, and conduction velocity over the nerve segment between the stimulation and the recording site.

Recently, the combination of electrodiagnostic evaluation, with the commonly used grasping test (reflex-based gross motor function) and the staircase test (skilled forelimb reaching), has been found to produce results with high translatability [65].

### 5.5. Biomechanical Analysis

Biomechanical analysis gives an objective evaluation of scar tissue formation and consists of measuring the force required to overcome the adhesion conjunctions between the nerve, scar tissue, and surrounding tissues. Different methods have been proposed, but the results obtained represent, with variability according to the precision of the utilized instruments, a quantitative expression of newborn scar tissue.

Dumanian [66] and his colleagues were the first to describe a method and device to measure the strength of nerve adhesion to surrounding muscles. They used a standard alligator clamp placed on the nerve and a force transducer connected, in turn, to a micrometer. The micrometer was distracted in 1 mm increments. The measurement is continued until final failure of the nerve or nerve pullout from the clamp.

Another method is to mount the nerve proximal stump on a digital force gauge using a suture connected to the load cell; then, the nerve is subjected to traction at a rate of 2 cm/min (or 1 cm/s) [24] until its complete detachment from the neural bed; the ultimate strength is recorded [57,67,68,69].

A different way consists of transecting both the proximal and distal ends of the nerve; the proximal end is then interconnected to a force transducer, which is connected, in turn, to a motorized drive with a constant extension rate of 29 mm/min. The force required to pull the nerve segment out of its tissue bed is recorded [10,22].

In another paper, after nerve and surrounding tissue removal from the animal, the distal end of the nerve was held by a clamp to the cross-head of an Instron machine. The subsequent cross-head movement (at a rate of 10 mm/min) then gradually peeled the nerve away from the adhesion site, and the maximum peeling force was recorded [15].

Finally, some recent papers have described a simple and cheap method that consists of connecting the nerve to a plastic can that is gradually filled with water at a constant flow of 100 mL/min. The adhesion force is obtained from the grams of water at the break moment [20,70,71].

### 5.6. Other Analysis

Other types of analyses have also been described, such as enzyme-linked immunosorbent assays (ELISAs) to evaluate neurothophic factor concentration [62], RNA and protein analysis [61], assessment of autotomy [37], functional analysis of the blood–nerve barrier and the perineurial barrier [69], and hydroxyproline and collagen assays [60].

In vitro culture of rat skin fibroblasts to test the efficacy of drug administration has also been described [58].

Histological staining can also be used to describe muscle tissue organization in order to evaluate atrophy and fibrous degeneration of the innervated muscles [13,49,57]. Moreover, atrophy is often investigated by measuring muscle wet weight. Finally, transmission electron microscopy has been adopted to describe the ultrastructure of nerve tissue and surrounding tissues [30,39,48,60,72].

## 6. How to Prevent Scar Formation? An Overview on Different Antiadhesion Devices

Every surgical practice on peripheral nerves is followed by postsurgical scar tissue formation. In order to limit this event, surgeons apply different procedures such as local or free tissue transfer and antiadherent items of different origins. There are many different kinds of antiadhesion devices, composed of different materials with different ways of application, but there is no evidence of their efficacies. Below is an overview of the different antiadhesion devices tested so far in experimental models.

### 6.1. Polysaccharide-Based Devices

Different polysaccharides were used as antiscarring agents, and the available preclinical studies on the polysaccharides-based devices are reported in Table 2.

#### 6.1.1. Hyaluronic acid

Hyaluronic acid is a glycosaminoglycan that is widely found in the body of all living organisms as it is an important extracellular matrix component. Since it does not exhibit species or tissue specificity and is biodegradable in vivo, it is often used as an ideal biomaterial. It has been demonstrated that hyaluronic acid reduces epineural and extraneural scar formation [36,38,67]. Additionally, biomechanical reduction of scar tissue has been documented [10], together with an improvement of latency [24].

#### 6.1.2. Carboxymethylcellulose

Carboxymethylcellulose is another biocompatible polysaccharide that acts as a physical barrier and can reduce scar formation in the central nervous system; it has been demonstrated that carboxymethylcellulose, in association with phosphatidylethanolamine, reduces peripheral nerve scarring and biomechanical resistance [67,71]. It has also been used in association with hyaluronic acid; additionally, in this case, it reduces scar formation, reduces inflammation cells and fibroblasts, and leads to better axonal organization [38,72], together with an increase in the quality of myelin sheets and the number of axons [49].

#### 6.1.3. Chitosan

Chitosan is a polysaccharide obtained by partial deacetylation of chitin. It has well-known advantageous properties, such as lack of toxicity and biocompatibility, biodegradability, and antimicrobial properties. Various forms of chitosan can be produced and microcrystallic chitosan gel applied to the proximal stump of a transected sciatic nerve has been shown to reduce the incidence and size of the neuroma and the formation of extraneural fibrosis [37]. It has also been used in the form of conduit in association with hyaluronic acid, and it has been demonstrated to reduce nerve scarring and promote nerve regeneration and recovery [48].

#### 6.1.4. Other polysaccharides

Few papers have tested other polysaccharides, such as oxidized regenerated cellulose [74], regenerating agent OTR4120 [22], cross-linked polysaccharides [28], or alginate [69,74].

Oxidized regenerated cellulose is a chemically altered form of cellulose used mainly as a hemostatic agent. It has been shown to not give an advantage to the prevention of nerve fibrosis; on the contrary, it interferes with healing by increasing inflammatory phenomena and granulomatous reactions [74]. Other polysaccharides have demonstrated their efficacy in the reduction of scarring through biomechanical and macro- and microscopical testing [22,28,69,74].

### 6.2. Collagen-Based Devices

The available preclinical studies on collagen-based devices are reported in Table 3.

The use of ADCON-T/N, a bioabsorbable gel composed of a polyglycan ester in a phosphate-buffered saline solution, showed a significant reduction of scar formation with no residual implant material [13,23,27]. Additionally, the use of collagen-based film wrapped around the suture stitches showed a reduction in epineural and perineural scar tissue formation [47,75,76]. Finally, a recent study showed that a collagen sheath derived from an acellular hypoallergenic dermal matrix wrapped around the suture leads to better nerve regeneration in terms of axon diameter [16].

### 6.3. Autologous Devices

Different autologous devices were used as antiadhesion devices, and the results of the preclinical studies are reported in Table 4.

#### 6.3.1. Amniotic Membrane

The amniotic membrane is the inner layer of fetal membranes; it is composed of an inner layer of epithelial cells on a thick basement membrane. It is nonimmunogenic, and it has been demonstrated to reduce inflammation, inhibit vascularization, combat infection, and reduce scarring. It is widely used in multiple fields of surgery and medicine, including skin substitute, wound care, urethral reconstruction, and repair of corneal and other tissues [77]. Its use in reducing peripheral nerve scarring has been demonstrated in different papers [14,39,40].

#### 6.3.2. Fat Grafting

In the last decade, adipose tissue has been widely studied in the field of regenerative medicine due to the presence of adipose-tissue-derived mesenchymal stem cells (which can differentiate into different cellular lineages) and its endocrine activity (release of adipocytokines, cytokines, transcriptional and growth factors). It is easy to access and harvest with painless procedures. The use of fat grafting in the prevention of peripheral scar tissue formation has had different results: it produces nerve stiffness reduction in biomechanical testing [66], but no significant differences were reported when compared to other antiadhesion devices. Moreover, in microscopical analysis, it appears to be able to reduce scar thickness [70].

#### 6.3.3. Vein Wrapping and Buccal Mucosa Graft

Another autologous tissue that has been tested for scar formation prevention is vein tissue, which is harvested from the same animal (femoral vein) and wrapped in a spiral pattern around the nerve [25] or harvested from the abdominal portion of the donor animal vena cava and wrapped around the ligated nerve [62]. In both cases, it has been demonstrated to reduce scar formation and improve nerve function recovery.

Finally, the use of a buccal mucosa graft has also been proposed as an antiadherent device since it is composed of nonkeratinized epithelium with underlying connective tissue and includes type I and III collagen. It has been shown that when wrapped around the nerve, it decreases adhesion and scar tissue formation but leads to higher inflammation in the early postoperative period [46].

### 6.4. Drugs

Several drugs have been tested; the preclinical results are reported in Table 5. Most of them (aprotinin, tacrolimus, mannose-6-phosphate, doxorubicin, mitomycin C, citicoline, cytidine-59-diphosphocholine-choline) were locally placed around the nerve [30,31,32,33,36,41,51,56]; others were intraepineurially injected (chondroitinase ABC) [61], intraperitoneally injected (citicoline and verapamil) [60,78], or intragastrically injected (tacrolimus) [58]. It has been shown that tacrolimus, an immunosuppressive drug used mainly after allogenic organ transplant, promotes nerve regeneration [79,80] and scar tissue reduction [36,58]. Moreover, the application of other drugs is correlated with scar formation reduction either in macro- or microscopical analysis. In addition, the improvement of axon quality has been reported in some papers, together with enhanced functional results.

### 6.5. Others

Many other devices/techniques have been investigated as antiscarring agents, and the preclinical results of these devices are reported in Table 6. A very recent study compared the efficacy of two novel biodegradable wraps made of synthetic 1% oxidized polyvinyl alcohol (OxPVA) and a leukocyte-fibrin-platelet membrane (LFPm) with the commercial product NeuraWrap, demonstrating their effectiveness in sustaining nerve regeneration, together with an absence of scar tissue/neuroma formation and significant inflammatory infiltrate [81].

A biodegradable polylactide (PLA) honeycomb film [83] and a nerve conduit composed of PLA and poly(e-caprolactone) (PCL) and enriched with hyaluronic acid [57] have been shown to prevent nerve adhesion. Additionally, a novel multilayer membrane made of PLA-based biodegradable polymer (E8002) containing L-ascorbic acid has been demonstrating to reduce scar formation compared to the same membrane without ascorbic acid [82]. Atkins et al. [52] proposed the local administration of neutralizing antibodies to TGF-β1 and TGF-β2, showing a significant reduction in intraneural scar formation. The use of Ankaferd blood stopper resulted in better healing and better results in the histopathological evaluations [59], and the use of an absorbable oxidized regenerated cellulose sheet showed the prevention of adhesion in a histological study [84]. Finally, low-dose radiation therapy [34] and early mobilization [15] have also been proposed.

## 7. Discussion

Traction neuropathies are diffused and frequent consequences of injuries or surgical procedures on peripheral nerves [2,7]. Surgeons and researchers have been trying to prevent scar tissue formation, especially by applying antiadhesion devices on the surgical site. Before human implantation, preclinical studies are of crucial importance for assessing the effectiveness of antiadhesion strategies; however, the results reported in the literature are not easily comparable due to the many different methods to induced scar formation as well as quantitatively evaluate the amount of scar tissue and its impact on peripheral nerve regeneration and function. The reason is connected to the absence of a shared, effective, reliable, reproducible, and standardized protocol to induce and test the scar tissue around the peripheral nerves [18,20,21,22].

The purpose of this review was, therefore, to resume and show the different strategies adopted in the last few years to simulate and evaluate scar tissue formation.

Different kinds of injuries have been proposed by researchers to simulate perineural scar tissue formation: nerve injury, injury to surrounding tissues, global injury by means of chemical or physical agents, and so on. Some authors have designed studies to simulate and evaluate perineural scars [17,18,19,20,21,22], but these works are incomplete because they have not considered all the aspects of induction and the evaluation methods available. Our review reveals that the widest protocol used to induce scar tissue is represented by section and suture of the nerve. This partially represents what really happens in clinical settings and results in a partial injury. In our opinion, an injury to the perineural tissue should always be associated in clinical settings by means of burning or chemical injury to the nerve. None of the papers have combined this kind of injury. It would, therefore, be very interesting and useful to test the combination of these methods in an experimental model that better mimics the clinic. Furthermore, researchers should consider that a different pattern of scar tissue arises if the nerve is transected or not. Without a nerve section and suture or without a crush injury, no internal scar will form, especially when there has been only a short period between the surgery and the analysis. In this way, no or only minimal impairment of nerve function will be present.

This aspect links to the other main aspect of this review: so many different types of analysis were performed to quantitatively or qualitatively assess the perineural scar and its reduction. We strongly encourage the use of quantitative analysis. Gross evaluation is certainly a fundamental step to macroscopically grading the scar, and the adoption of a numerical grading scheme is necessary to quantify (or semiquantify) the amount of scar tissue. Different grading schemes have been adopted [13,14,17,23,27,28] and most of the authors have used these schemes to score scar tissue during macroscopical inspection. Nevertheless, other authors have described only the presence or absence of scar tissue, and, sometimes, they qualitatively describe the scar tissue by relying on subjective observations.

Microscopical evaluation is usually conducted both on the nerve and scar tissue. Beyond the type of staining used, the quantitative evaluation of the perineural scar is very important. First of all, a staining solution that is able to visualize collagen fibers specifically (such as Masson’s Trichrome, Sirius Red) is preferred to the classical H&E and methylene blue stainings. Quantification of scar tissue is proposed in different ways, but the most adopted is the calculation of the scar tissue formation index (by dividing the mean thickness of the scar tissue by the mean thickness of the nerve tissue) [18,28,30,31,32,33,34,36,37,38,39,40,41]. Moreover, in these cases, some other authors have used different parameters to quantify (or semiquantify) the scar tissue histologically; sometimes, the structure of scar tissue is only qualitatively described. Some authors have added immunohistochemistry to conventional staining. Very interesting are the attempts to detect scar tissue using antibodies against macrophages and lymphocytes, but it is difficult to obtain a quantification of these findings [21,37,59].

Biomechanical analysis provides another quantitative parameter and consists of measuring the peak force required to pull the nerve from the muscular bed. Different tools have been adopted, and, in general, they consist of applying a continuous force to the nerve until the complete detachment of the nerve from the surrounding tissue. This should be the other main method that is useful to demonstrate the strength of the scar tissue in preclinical analysis. In addition, the structure and function of the nerve must be assessed. Quantification of different parameters (number of fibers, axon and fiber diameter, myelin thickness) are important to assess the degree of nerve regeneration and can be related to electrophysiological parameters and functional evaluations, often assessed in studies dealing with peripheral nerve scarring. In our thoughts, morphological and functional impairments correlate more to a direct nerve injury than an experimental perineural scar. Hence, morphometry and electrophysiology should be associated, especially when nerve injury and repair have been performed or when scar neuropathy has lasted for several months.

Different animal models have been proposed, each one with pros and cons. Mice are easy to house and allow quick and easy surgery. The sciatic nerve allows the performance of all the main analyses described above. Otherwise, in this model of nerve repair, functional and electrophysiological tests are more difficult to perform due to the smallness of the animal itself. Conversely, rats allow the testing of both the sciatic nerve and the median nerve; the size of these nerves is feasible for nerve repair and electrodiagnostic tests. Furthermore, functional evaluation on the median nerve can be carried out, especially when a direct nerve injury is performed.

Due to the limitations of the present models, no definitive conclusion can be derived about the efficacy of antiadhesion devices. In most of the experiments, every treated group showed scar reduction according to the evaluation methods. The most employed antiadhesion devices were polysaccharide-based and collagen-based ones. Their effectiveness was, in most cases, well known in spine surgery, tendon surgery, or abdominal surgery. It is similar for biological barriers: vein wrapping has been described to protect nerve sutures in the past [25,85], with good clinical outcomes. Fat grafts were previously adopted in spine surgery with controversial results; in peripheral nerves, it seems to be effective, and promising results were obtained with Coleman’s lipoaspirate [70]. From our review, it has emerged that the application of amniotic membrane can be promising, considering the increased chance of tissue storage [14,39,40]. This requirement can also be considered a limit for this technique. Drugs and other devices can also be promising, but currently, no clinical experience exists.

This preclinical literature review suggests that we reconsider the whole argument of traction neuropathies. This pathology was classified by Millesi [86] in intra- and extraneural scar, but more extensive classification would be effective to better understand the correct treatment. Perineural scarring arises after closed trauma and nerve decompression or can be associated with a repaired nerve. The intraneural involvement is different in each case, and different approaches should be considered. When an intraneural scar is present, it should be treated as a neuroma in continuity. In contrast, when the perineural scar is the main concern, other procedures should be performed. According to our clinical practice and the results obtained in these experimental models, vein wrapping can be a feasible procedure to prevent intraneural and epineural adhesion after nerve suture. Otherwise, in a secondary peripheral nerve decompression, the application of an antiadhesion device, either in the form of gel or film composed of polysaccharide or collagen, could be adequate as well as autologous lipoaspirate, local adipose flap, synovial flap, or amniotic membrane wrapping. The choice, at state of the art, is up to the individual surgeon’s experience and availability since no clear evidence exists.

To better understand traction neuropathies, a more extensive classification should be designed by considering the extension of the scar, the amount of fibrous tissue (both pre- and intraoperative by means of a quantitative scale, as proposed by Petersen [27]), and previous surgery on the nerve (suture, traction injury, nerve decompression). On these, prognostic criteria would be found and a more fitted treatment protocol developed.

In our experience and considering the literature, to completely evaluate scar tissue formation around a nerve, we require a scored macroscopical analysis, a quantitative microscopical analysis conducted with a staining solution that is able to collagen fibers visualize specifically (such as Masson’s Trichrome, Sirus Red) in order to clearly measure the thickness and extension of scar tissue, and a computed biomechanical analysis with the appropriate microinstruments. The structure, organization, and function of the nerve should also accompany scar tissue data, but these are not always mandatory since they depend on the type of nerve injury induced.

Finally, this review reports on the different antiadhesion devices that have been experimentally tested so far. Due to the high variability of scar induction and evaluation methods described, it is not possible to compare the results obtained in terms of scar reduction and efficacy of the antiadhesion devices employed.

## Figures and Tables

**Table 1:** No antiscarring agents tested.

Reference	Method to Induce Scar Formation	Animal and Nerve Model	Analyses	Results
Lemke et al., 2017 [21]	Application of “glutaraldehyde glue” on the nerve and surrounding muscle or scratching	Female Sprague–Dawley Rat Sciatic nerve	- Functional evaluation (CatWalk, SFI) (once a week) - Gross evaluation (Petersen’s classification) (3 weeks) - Histology on nerve and surrounding tissue (H&E, Masson’s trichrome and Luxol fast blue, chromotrop-aniline-blue) (3 weeks) - IHC (2F11, S100, CD-68, CD-3, CD-8) (3 weeks) - Electrophysiological evaluation (3 weeks)	Severe intra- and perineural scarring, vigorous nerve inflammation and nerve degeneration and functional deficit.
Crosio et al., 2014 [20]	Burning or scratching	Male Mouse Sciatic nerve	- Biomechanical analysis (3 weeks) - Histology on nerve and surrounding tissue (Picrosirius staining) (3 weeks)	Both methods produced fibrotic reactions with no differences in biomechanical results between the two methods; histology showed a different distribution pattern of the scar tissue.
Okuhara Y et al., 2014 [19]	Irradiation of the nerve with X-radiation	Female LEW/CrlCrlj Rat Sciatic nerve	- Functional evaluation (SFI) (4, 8, 12, 16, 20, and 24 weeks) - Electrophysiological evaluation (24 weeks) - Gross evaluation (qualitative) (24 weeks) - Histology on nerve and surrounding tissue (Masson’s trichrome and Toluidine Blue for morphometric analysis) (24 weeks)	Scar formation around the radiated nerve. No differences in SFI between groups, but axonal degeneration in the irradiated nerve.
Zanjani et al., 2013 [18]	Laceration, crush, mince, and burn of the surrounding muscles	Female Wistar Rat Sciatic nerve	- Functional evaluation (Toe Out Angle) (weekly up to 4 weeks) - Gross Evaluation (Petersen’s classification) (1, 2, 3, 4 weeks) - Histology on nerve and surrounding tissue (Masson’s trichrome) (1, 2, 3, 4 weeks)	Scar tissue formation surrounding the sciatic nerve in gross examination and histological analysis; no differences in functional assessment compared to control.
Abe et al., 2005 [17]	Nerve bed cauterization and suturing the nerve in place	Male Japanese White Rabbit Sciatic nerve	- Gross evaluation (classification based on Fontana’s bands and Sakurai’s classification) (6, 14, 22, 30, or 38 weeks) - Electrophysiological evaluation (6, 14, 22, 30, or 38 weeks) - Nerve fascicle blood flow (6, 14, 22, 30, or 38 weeks) - Histology on nerve and surrounding tissue (Toluidine Blue) (6, 14, 22, 30, or 38 weeks)	Adhesion of peripheral nerve to surrounding tissues results in fibrosis in the nerve. Compound muscle action potentials were reduced in amplitude, and blood flow was significantly decreased at adhesion sites in Group IIb.

SFI (Sciatic functional index), IHC (Immunohistochemistry), 2F11 (antibody labelling neurofilament), S100 (antibody labelling Schwann cells), CD-68 (Cluster of Differentiation 68, antibody labelling macrophages), CD-3 (Cluster of Differentiation 3, antibody labelling T cells), CD-8 (Cluster of Differentiation 8, antibody labelling cytotoxic T cells).

**Table 2:** Polysaccharide-based antiscarring agents.

Reference	Method to Induce Scar Formation	Agent	Animal and Nerve Model	Analyses	Results
Hachinota et al., 2020 [73]	Section of transvers carpal ligament, excision of median nerve bed, suture of carpal ligament	Alginic acid-based gel formulation	Japanese White Rabbit Median nerve	- Electrophysiological evaluation (1, 2, 3, 6 weeks) - Macroscopic evaluation (adhesion scoring system modified from Palatinsky ones) - Histology on tissues excised after electrophysiological evaluation (H&E staining)	Longer latency, not significant, in the control group. Lower adhesion score values in the treatment group at 2–3–6 weeks, more scar tissue in the control group. More severe perineural fibrosis in the control group.
Li et al., 2018 [48]	Crush injury	Chitosan conduit (CC); hyaluronic acid (HA); CC + HA	Sprague–Dawley RatSciatic nerve	- Functional evaluation (SFI) (4, 8, 12 weeks) - Gross Evaluation (Petersen’s classification) (4, 8, 12 weeks) - Electrophysiological evaluation (12 weeks) - Histology on nerve and surrounding tissue (H&E, Masson’s trichrome and Toluidine Blue) (4, 8, 12 weeks) - Ultrastructural evaluation (TEM) (4, 8, 12 weeks)	Both chitosan and HA inhibited extraneural scarring, promoted nerve regeneration, increased nerve conduction velocity, and improved the recovery of nerve function.
Mekaj et al., 2017 [36]	Section + suture	HA	Male European Rabbit Sciatic nerve	- Gross evaluation (Petersen’s classification) (12 weeks) - Muscle wet weight (12 weeks) - Histology on nerve and surrounding tissue (H&E, Masson’s trichrome) (12 weeks) - Nerve IHC (S100) (12 weeks)	Reduction scar around the nerve, both macroscopically and microscopically. Increased nerve diameter. Higher gastrocnemius mass. Improved microstructural organization. Higher expression of S100.
Tos et al., 2016 [71]	Burning	Carboxymethylcellulose (CMC)-polyethylene oxide (PEO) gel	CD1 Mouse Sciatic nerve	- Biomechanical analysis (3 weeks) - Histology on nerve and surrounding tissue (Picrosirius staining) (3 weeks)	Reduction in scar tissue after CMC–PEO gel application. The qualitative histological analysis supported the biomechanical findings depicting the pattern of scar tissue.
Urano et al., 2016 [68]	Enwrapping with silicon tube (nerve compression)	CMC-phosphatidyl-ethanolamine (PE)	Male Lewis RatSciatic nerve	- Electrophysiological evaluation (1, 2, 3 months) - Biomechanical analysis (1, 2, 3 months) - Muscle wet weight (1, 2, 3 months) - Histology on nerve and morphometric analysis (Toluidine Blue) (1, 2, 3 months)	Electrophysiology showed significantly quicker recovery; mean wet muscle weight was constantly higher; the axon area at one month was twice as large as control.
Marcol et al., 2011 [37]	Section + suture	Chitosan	Male Wistar Rat Sciatic nerve	- Autotomy assessment (daily, until 20th week) - Histology on nerve and surrounding tissue (H&E, Masson’s trichrome and Toluidine Blue) (20 weeks) - Nerve IHC (CD-68, GAP43) (20 weeks)	High incidence of amputations (about 100%, no sig. diff.). Reduction in microscopical analysis of neuroma in chitosan; significant reduction of scar around nerve; increased mast cells and macrophages in chitosan.Application of the microcrystalic chitosan gel is easy and requires no special equipment but does not influence the features of neuropathic pain.
Park et al., 2011 [38]	Section + suture	HA-CMC	Sprague–Dawley Rat Sciatic nerve	- Gross evaluation (Petersen’s classification) (3, 6, 9, 12 weeks) - Histology on nerve and surrounding tissue (H&E, Masson’s trichrome) (3, 6, 9, 12 weeks)	Macroscopical scar reduction. Reduction of inflammation cells and fibroblasts. Reduction of scar formation index. Better axonal organization.
Hernández-Cortés et al., 2010 [74]	Tissue aggression (cauterization of muscle bed)	Oxidized regenerated cellulose	Male Sprague-Dawley RatSciatic nerve	- Histology on nerve and surrounding tissue (H&E, Masson’s trichrome, PAS, or Syrius red) (3 and 6 weeks)	No statistical differences in intra- and perineural scars, which demonstrate no antifibrogenic effect of oxidized regenerated cellulose. Inflammatory phenomena and foreign body granulomatous reactions were more frequently detected in oxidized regenerated cellulose-treated samples.
Yamamoto et al., 2010 [67]	Burning muscle + epi- and perineurium removal	CMC–PE	Lewis Rat Sciatic nerve	- Gross evaluation (evaluation of healing of skin and fascia—Grade 1–3) (6 weeks) - Biomechanical analysis (6 weeks) - Histology on nerve and surrounding tissue (H&E Masson’s trichrome) (1, 2, 3, 4, 5, 6 weeks) - Electrophysiology (2,7, 20, and 42 days) - Muscle wet weight (2,7, 20, and 42 days)	CMC–PE hydrogel offered superior efficacy to 1% HA and caused no delay in wound healing. Reduction of macroscopical scar, reduction of scar in biomechanical testing. Electrophysiological and muscle weight analyses demonstrated the effectiveness of CMC–PE treatment after extensive internal neurolysis.
		HA			
Magill et al., 2009 [72]	Section + suture	HA-CMC (Seprafilm)	Male Lewis Rat Sciatic nerve	- Functional evaluation (SFI) (biweekly until day 32) - Histology on nerve and surrounding tissue and morphometric analysis (Toluidine Blue) (18, 32, 42 days) - Ultrastructural evaluation (TEM) (18, 32, 42 days)	Qualitatively less perineural scar tissue was observed using Seprafilm. No functional or histological deleterious effects were detected with Seprafilm placed on intact nerves or cut and repaired nerves.
Zuijdendorp et al., 2008 [22]	Crush injury	Regenerating agents (sulfated glycosaminoglycan)	Female Wistar RatSciatic nerve	- Biomechanical analysis (6 weeks) - Magnetoneurography (5 weeks) - Footprint analysis (1, 7, 14, 17, 21, 24, 28, 35 and 42 days)	Reduction of biomechanical resistance. No differences in magnetoneurography and functional analysis were detected.
Dam-Hieu et al., 2005 [28]	Abrasive injury/section + suture	Auto cross-linked polysaccharide (ACP) with different viscosity	Male Sprague–Dawley Rat Sciatic nerve	- Gross evaluation (4-point qualitative Scale) (4 weeks) - Histology on nerve and surrounding tissue (H&E, Masson’s trichrome, Gordon and Sweets stain and Toluidine Blue) (4 weeks)	Significant reduction of scar tissue formation was observed through macro and micro analyses.
Ohsumi et al., 2005 [69]	Burning	Alginate sol	Lewis Rat Sciatic nerve	- Histology on nerve and surrounding tissue (H&E Masson’s trichrome) (1, 2, 3, 4, 5, 6 weeks) - Functional analysis of the blood–nerve barrier and the perineurial barrier (6 weeks) - Biomechanical analysis (6 weeks)	Strong inhibition of perineurial granulation, recovering of the perineurial barrier function, antiadhesive effect.
Smit et al., 2004 [10]	Section + suture	HA	Female Wistar Rat Sciatic nerve	- Biomechanical analysis (6 weeks)	Significant biomechanical reduction of adhesion after HA application.
Ikeda et al., 2003 [24]	Burning muscular bed + suture	HA (after 6 weeks)	White Japanese RabbitSciatic nerve	- Electrophysiological evaluation (6 weeks) - Histology on nerve and surrounding tissue (Masson’s trichrome) (6 weeks) - Biomechanical analysis (6 weeks)	Significant latency reduction. Qualitative reduction of scar in microscopical analysis. No significant reduction in biomechanical analysis.
Ozgenel et al., 2003 [35]	Section + suture	HA	Male Sprague–Dawley Rat Sciatic nerve	- Functional evaluation (SFI) (every two weeks until week 12) - Gross evaluation (Petersen’s classification) (4, 12 weeks) - Electrophysiological evaluation (12 weeks) - Wet muscle weight (12 weeks) - Histology on nerve and surrounding tissue (Masson’s trichrome 4, 12 weeks, H&E, Weil method for morphometric analysis) (12 weeks)	Significant reduction in scarring, better conduction velocities, increased axon and fiber diameter, and faster functional recovery.
Adanali et al., 2003 [49]	Section + suture	HA–CMC	White New Zealand Rabbit Sciatic nerve	- Gross evaluation (qualitative) (3 months) - Electrophysiological evaluation (3 months) - Histology on muscle (H&E, Masson’s trichrome) (3 months) - Histology on nerve and surrounding tissue (H&E, Masson’s trichrome, Toluidine Blue) (3 months)	Macroscopically reduction of scar tissue around the nerve. Increased quality of myelin sheets and the number of axons.

SFI (Sciatic functional index), TEM (Transmission Electron Microscope), IHC (Immunohistochemistry).

**Table 3:** Collagen-based antiscarring agents.

Reference	Method to Induce Scar Formation	Agent	Animal and Nerve Model	Analyses	Results
Colonna et al., 2019 [16]	Section + suture	Collagen sheath derived from an acellular hypoallergenic dermal matrix (OrACELL)	Female Wistar Rat Median nerve	- Functional evaluation (grasping test) (every two months) - Histology on nerve and surrounding tissue and morphometric analysis (Toluidine Blue) (7 months)	Axon diameter was higher in the treated group. No significant differences in the functional test were observed.
Lee et al., 2014 [76]	Section + suture	Collagen-based film (NeuraGen)	Male Wistar Rat Sciatic nerve	- Electrophysiological evaluation (12 weeks) - Wet muscle weight (12 weeks) - Histology on nerve and surrounding tissue and morphometric analysis (Toluidine Blue) (12 weeks)	Reduction of scar in microscopical analysis, although the scar-decreasing effect of bioabsorbable nerve wrap did not translate into a better motor nerve recovery.
Mathieu et al., 2012 [47]	Section + suture	Collagen membrane and vein wrapping	Female Wistar Rat Sciatic nerve	- Gross evaluation (Petersen’s classification) (12 weeks) - Histology on nerve and surrounding tissue (Masson’s trichrome—extraneural and intraneural fibrosis, foreign body reaction)	The collagen membrane was effective in reducing neural scar formation. Autologous vein wrapping also showed a favorable effect in this indication despite less successful histological outcomes.
Kim et al., 2010 [75]	Section + suture	Collagen wrap	Sprague–Dawley Rat Sciatic nerve	- Gross evaluation (Petersen’s classification) (3 months) - Histology on nerve and surrounding tissue and morphometric analysis (Toluidine Blue) (3 months)	Significant reduction of inner epineurium thickness in the treated group.
Isla et al., 2003 [13]	Section + suture or repair with silastic tube	ADCON/TN	Male Wistar Rat Ulnar nerve	- Gross evaluation (4-point qualitative Scale) (3 months) - Histology on nerve, surrounding tissue and muscle (H&E, Masson’s trichrome) (3 months)	Significant reduction of fibrosis. No differences in terms of fiber density.
Palatinsky et al., 1997 [23]	Scratch; a second neurolysis performed 4 weeks later	ADCON/TN (applied after the second neurolysis)	Sprague Dawley Rat Sciatic nerve	- Gross evaluation (4-point qualitative Scale) (4 and 8 weeks) - Histology on nerve and surrounding tissue (H&E, Masson’s trichrome, phenylenediamine staining) (4 and 8 weeks)	Significant reduction of composite score (macroscopical evaluation). No statistical difference in axons diameter.
Petersen et al., 1996 [27]	External neurolysis, abrasive injury on muscle and nerve, section + suture	ADCON/TN	Lewis’s Albino Rat Sciatic nerve	- Gross Evaluation (Petersen’s classification) (4 and 6 weeks) - Electrophysiological evaluation (4 and 6 weeks) - Histology on nerve and surrounding tissue (H&E, silver stain, van Gieson’s stain, Toluidine blue for morphometric analysis) (4 and 6 weeks)	Significant reduction of scar tissue; no differences in morphometrical analysis.

**Table 4:** Autologous tissues used as antiscarring agents.

Reference	Method to Induce Scar Formation	Agent	Animal and Nerve Model	Analyses	Results
Cherubino et al., 2017 [70]	Burning	Fat Graft	CD1 nude Mouse Sciatic nerve	- Biomechanical analysis (4 weeks) - Histology on nerve and surrounding tissue (Picrosyrius red) (4 weeks)	No significant difference in biomechanical analysis. Reduction of scar observed through microscopical analysis
Baltu et al., 2017 [46]	Epineurectomy	Buccal mucosa graft	Female Sprague-Dawley Rat Sciatic nerve	- Gross evaluation (Petersen’s classification) (4, 8 weeks) - Histology on nerve and surrounding tissue (H&E, Masson’s trichrome—extraneural scar tissue and inflammation) (4, 8 weeks)	Buccal mucosa graft decreases postoperative adhesion and scar tissue formation. Higher inflammation at 4 weeks.
Murakami et al., 2014 [62]	Ligature on sciatic nerve	Vein Wrapping	Male Wistar Rat Sciatic nerve	- Gross evaluation (qualitative evaluation) (14 days and 5 months) - Functional evaluation (von Frey filaments at 1, 4, 7, 14, 21, 28 days; CatWalk system, first 2 weeks) - IHC on L4-L5 DRG (CGRP, ATF3) (14 days) - Histology on nerve and surrounding tissue (H&E, Toluidine Blue) (14 days and 5 months) - ELISA assay on nerve tissue (NGF, VEGF, and HGF) (1, 3, 7, 14, 28 days).	Significant allodynia reduction. Significant increase in VEGF and HGF. Reduction of immunoreactive cells in dorsal root ganglia.
Meng et al., 2011 [39]	Section + suture	Amniotic membrane	Male Sprague–Dawley Rat Sciatic nerve	- Functional evaluation (SFI) (weekly) - Gross evaluation (Petersen’s classification) - Electrophysiological tests (every 4 weeks until Week 12) - Histology on nerve and surrounding tissue (Picrosyrius red, toluidine blue for morphometrical analysis) (4, 8, 12 weeks) - Ultrastructural evaluation (TEM) (4, 8, 12 weeks)	Significant reduction of scar index. No functional and morphological differences were observed.
Kim et al., 2010 [14]	Section + suture	Amniotic membrane	White New Zealand Rabbit Ulnar nerve	- Gross evaluation (4-point qualitative Scale) (3 months) - Histology on nerve and surrounding tissue (Masson’s trichrome—morphometrical analysis) (3 months)	Four-point evaluation system was significant in the treatment group. Significant reduction of scar thickness.
Ozgenel et al., 2004 [40]	Epineurectomy	Amniotic membrane + HA	Male Sprague–Dawley Rat Sciatic nerve	- Gross evaluation (Petersen’s classification) (4 and 12 weeks) - Histology on nerve and surrounding tissue (H&E) (4 and 12 weeks)	Significant reduction in scarring was observed through microscopical analysis.
Xu et al., 2000 [25]	Silastic tube around the nerve	Vein wrapping (after 8 months from nerve compression)	Sprague–Dawley Rat Sciatic nerve	- Functional evaluation (SFI) (4, 8, 12, 24, and 48 weeks) - Gross Evaluation (qualitative evaluation) (4, 8, 12, 24, and 48 weeks) - Electrophysiological tests (4, 8, 12, 24, and 48 weeks) - Histology on nerve and surrounding tissue (H&E, Masson’s trichrome, silver staining, toluidine blue) (4, 8, 12, 24, and 48 weeks)	Significant improvement in functional analysis. Electromyography and microscopical analysis showed no significant scar reduction.
Dumanian et al., 1999 [66]	Epineurectomy	Fat graft	Sprague–Dawley Rat Sciatic nerve	- Biomechanical analysis (2 months) - Histology on nerve and surrounding tissue (Masson’s trichrome) (2 months)	Significant reduction of nerve stiffness in biomechanical analysis. Insignificant reduction of scar thickness in microscopical analysis. Higher but not significant incidence of neuropathy in fat-graft group.

SFI (Sciatic functional index), TEM (Transmission Electron Microscope), IHC (Immunohistochemistry), H&E (Hematoxylin Eosin staining), CGRP (Calcitonin gene related peptide), ATF3 (Activating transcription factor 3), VEGF (vascular endothelial growth factor), HGF (Hepatocyte growth factor), NGF (Nerve growth factor).

**Table 5:** Drugs used as antiscarring agents.

Reference	Method to Induce Scar Formation	Agent	Animal and Nerve model	Analyses	Results
Mekaj et al., 2017 [36]	Section + suture	Tacrolimus (FK506)	Male European Rabbit Sciatic nerve	- Gross evaluation (Petersen’s classification) (12 weeks) - Muscle wet weight (12 weeks) - Histology on nerve and surrounding tissue (H&E, Masson’s trichrome) - Fibroblast and inflammatory cell counts (12 weeks) - Nerve IHC (S100) (12 weeks)	Scar reduction around the nerve, both macroscopically and microscopically. Increased nerve diameter. Higher gastrocnemius mass. Improved microstructural organization. Higher expression of S100.
Zhu et al., 2017 [61]	Silicone tube around the nerve	Decompression and chondroitinase ABC (6 weeks after compression injury)	Male Sprague–Dawley Rat and Male C57BL/6 Mouse Sciatic nerve	- Electrophysiology (1 month) - RNA (neuron-glial antigen 2, phosphacan, brevican, versican, aggrecan, and decorin) and protein expression (decorin, aggrecan, laminina 2, collagen IV, and fibronectin) (1 month) - Nerve IHC (decorin, aggrecan, laminina 2, collagen IV, and fibronectin) (1 month)	Surgical decompression alone does not reverse the functional changes to the nerve, whereas the administration of chondroitinase-ABC, in addition to decompression, resulted in functional improvement.
Vural et al., 2016 [41]	Abrasion	Mitomycin C/ Daunorubicin	Male Wistar Rat Sciatic nerve	- Gross evaluation (Petersen’s classification) (8 weeks) - Histology on nerve and surrounding tissue (H&E and Masson’s trichrome—fibroblast count) (8 weeks)	Macroscopically, mitomycin C, and daunorubicin decreased adhesion. Scar tissue thickness and fibroblast/fibrocyte cell number were reduced.
Xue et al., 2016 [60]	Section + suture	Verapamil (calcium channel blockers)	Sprague–Dawley Rat Sciatic nerve	- Gross evaluation (qualitative evaluation) (4 and 12 weeks) - Nerve IHC (collagen I) (4 and 12 weeks) - Ultrastructural evaluation (TEM) (4 and 12 weeks) - Hydroxyproline and collagen assay (4 and 12 weeks)	The collagen content of nerve scar was apparently less than that of the control group; more cytoplasmic vesicles in the fibroblasts of the treated group were observed.
Kaplan et al., 2014 [78]	Section + suture	Citicoline	Female Wistar Albino Rat Sciatic nerve	- Functional evaluation (SFI) (4, 8, 12 weeks) - Electromyography (12 weeks) - Gross evaluation (Petersen’s classification) (12 weeks) - Histology on nerve and surrounding tissue (Masson’s trichrome and Toluidine Blue for morphometrical analysis) (12 weeks)	Improvement of SFI. Significant reduction in scarring. Significant increase in myelinated axons in C900 and reduction of scar in the treated group.
Que et al., 2013 [58]	Section + suture	Tacrolimus (FK506)	Male Sprague–Dawley Rat Sciatic nerve	- Nerve IHC (TGF-β) (4 weeks) - Histology on nerve and surrounding tissue (Masson’s trichrome) (4 weeks) - In vitro analysis	FK506 has a valid effect on scar formation reduction in sciatic nerve-injured rat by inducing fibroblast apoptosis.
Ngeow et al., 2011 [50]	Section + suture	Triamcinolone acetonide, Interleukin-10 (IL 10), mannose-6-phosphate (M6P)	C57 Black-6 Mouse Sciatic nerve	- Electrophysiological evaluation (6 and 12 weeks) - Functional evaluation (CatWalk) (1, 3, 6, 9, and 12 weeks - Histology on nerve and surrounding tissue (Picrosirius staining) (6 and 12 weeks)	The percentage of scarring was not significantly different between methods in microscopical analysis. Reduction of compound action potential in triamcinolone and M6P 200 was observed through EMG.
Ngeow et al., 2011 [51]	Section + suture	Mannose-6-phosphate	C57 Black-6 mice Sciatic nerve	- Electrophysiological evaluation (6 and 12 weeks) - Histology on nerve and surrounding tissue (Picrosirius staining) (6 and 12 weeks) - Functional evaluation (CatWalk) (1, 3, 6, 9 and 12 weeks	Larger compound action potential and better functional recovery in early evaluation. Reduction in collagen staining.
Aslan et al., 2011 [56]	Section + suture (immediate or 3 days later)	CDP-choline, cytidine, choline, or cytidine–choline (during nerve repair)	Female Sprague–Dawley Rat Sciatic nerve	- Functional evaluation (SFI) (4, 8, 12 weeks) - Gross evaluation (Petersen’s classification) (12 weeks) - Histology on nerve and surrounding tissue (H&E, Weil method for morphometrical analysis) (12 weeks)	Treatment with CDP-choline or cytidine–choline reduced scar formation and decreased nerve adherence.
Albayrak et al., 2010 [30]	Abrasion	Doxorubicin	Male Wistar Albino Rat Sciatic nerve	- Gross evaluation (Petersen’s classification) (12 weeks) - Histology on nerve and surrounding tissue (Toluidine Blue) (12 weeks) - Ultrastructural evaluation (TEM) (12 weeks)	Topical application of doxorubicin effectively reduced epineural scar formation.
Atkins et al., 2007 [53]	Section + suture	IL-10	C57 Black-6 MouseSciatic nerve	- Electrophysiological analysis (6 weeks) - Histology on nerve and surrounding tissue (Picrosirius staining and Toluidine Blue for morphometrical analysis) (6 weeks)	Compound action potential and area of staining for collagen not significantly different compared to controls. Higher number of myelinated fibers compared to control but no difference with the other groups.
Ozay et al., 2007 [31]	Section + suture	Citicoline	Female Sprague-Dawley Rat Sciatic nerve	- Functional evaluation (SFI) (4, 8, 12 weeks) - Electrophysiological analysis (4, 12 weeks) - Gross evaluation (Petersen’s classification) (4 weeks) - Histology on nerve and surrounding tissue (Masson’s trichrome, H&E, Weil method for morphometrical analysis) (4, 12 weeks)	Rats treated with citicoline showed significantly better SFI and improvement at 12 weeks of electromyography. Nerves were surrounded by only a very thin, lucent membrane and showed thin dark bands of connective tissue surrounding the nerve.
Ilbay et al., 2005 [32]	Scratch	Mitomycin C	Male Wistar Rat Sciatic nerve	- Gross evaluation (Petersen’s classification) (4 weeks) - Histology on nerve and surrounding tissue (H&E, Masson’s trichrome— fibroblasts/fibrocytes count) (4 weeks)	Macroscopical and microscopical reduction of perineural adhesions in the treated groups; lower number of fibroblast/fibrocytes.
Gorgulu et al., 1998 [33]	External neurolysis, abrasive injury, anastomosis	Aprotinin	Male Sprague-Dawley RatSciatic nerve	- Functional analysis (sciatic nerve function) (weekly) - Gross evaluation (Petersen’s classification) (4, 6 weeks) - Histology on nerve and surrounding tissue (Masson’s trichrome) (4, 6 weeks)	Scar reduction after aprotinin application. No differences in neurological tests were observed.

**Table 6:** Other antiscarring agents.

Reference	Method to induce scar formation	Agent	Animal and Nerve model	Analyses	Results
Kikuchi et al., 2020 [82]	Burning	Polylactic acid (PLA)-based biodegradable three-layered membrane (E8002) with or without L-ascorbic acid (AA)	Male Sprague–Dawley Rat Sciatic nerve	- Motor functional (rotarod) and mechanical sensitivity (von Frey) evaluation (before surgery and 2, 4, 6 weeks after). - Gross evaluation (Petersen’s classification) (6 weeks) - Histology on nerve and surrounding tissue (aldehyde fuchsin Masson–Goldner staining) (6 weeks)	AA in E8002 has an antiadhesional effect by enhancing fibrinolysis. Adhesion formation was lower in the group containing AA. Motor function and mechanical sensitivity were not impaired after surgery, and no differences were detected among groups.
Stocco et al., 2019 [81]	Section + suture	Wraps made of a synthetic 1% oxidized polyvinyl alcohol (OxPVA) and a leukocyte-fbrin-platelet membrane (LFPm) compared to NeuroWrap	Sprague–Dawley rats Sciatic nerve	- Functional analysis (sciatic function index assessment) (2 and 12 weeks) - Gross evaluation (12 weeks) - Histology on nerve and surrounding tissue (H&E, IHC and Toluidine blue) (12 weeks) - Ultrastructural analysis (TEM) (12 weeks) - Neural collagen deposition evaluation (12 weeks)	LFPm wraps were completely resorbed, while residues of OxPVA and NeuraWrap were observed. Functional recovery was achieved in all groups. Additionally, at the morphological level, scar tissue formation and inflammatory infiltrate were not observed. Both myelinic and unmyelinic axons were observed.
Shintani et al., 2018 [57]	Burning	Polylactide (PLA)-poly(e-caprolactone) PCL conduit and HA	Lewis Rat Sciatic nerve	- Gross evaluation (Petersen’s classification) (6 weeks) - Biomechanical examination (6 weeks) - Electrophysiological evaluation (6 weeks) - Muscle wet weight and histology (6 weeks) - Nerve IHC (antineurofilament, anti-CD68, and anti-CCR7) (6 weeks)	Morphological properties of axons were preserved with PLA-PCL conduit. HA was less effective for nerve protection from adhesion.
Servet et al., 2016 [59]	Section + suture	Ankaferd blood stopper (ABS) hemostatic agent	Male Sprague–Dawley Rat Sciatic nerve	- Electrophysiology (12 weeks) - Histology on nerve and surrounding tissue (H&E, Masson’s trichrome, and Luxol fast blue) (24 weeks) - Nerve IHC (CD68) (24 weeks)	Significant improvement of latency and speed in the ABS group. Other results were not statistically different.
Okui et al., 2010 [83]	Neurolysis and burning	PLA	Male Lewis Rat Sciatic nerve	- Electrophysiological evaluation (6 weeks) - Histology on nerve and surrounding tissue (H&E, Masson’s trichrome) (1, 2, 4, 6 weeks) - Muscle wet weight (2, 4, 6 weeks) - Analysis of the blood–nerve barrier (2 days)	PLA film has the potential to prevent adhesion even after internal neurolysis, and it is a useful substitute for perineurium.
Atkins et al., 2006 [52]	Section + suture	TGF-β1and TGF-β2	Male Sprague–Dawley Rat Sciatic nerve	- Electrophysiological evaluation (7 weeks) - Histology on nerve and surrounding tissue (Picrosirius staining and Toluidine Blue for morphometrical analysis) (7 weeks)	No differences in the percentage of collagen staining area were observed. Compound action potential ratios significantly smaller; increased number of myelinated fibers distally (no differences between TGF-β1 and TGF-β2)
Gorgulu et al.,2003 [34]	Neurolysis vs. scratching vs. suture vs. radiation treatment	Low-dose radiation therapy (24 h after surgery)	Male Sprague–Dawley Rat Sciatic nerve	- Functional analysis (sciatic nerve function) (weekly) - Gross evaluation (Petersen’s classification) (6 weeks) - Histology on nerve and surrounding tissue (Masson’s trichrome—fibroblast/fibrocytes count) (6 weeks)	Significant reduction of scar tissue in radiation + surgery groups. No increase in scar tissue formation after radiation in normal nerves was observed.
Ikeda et al., 2002 [84]	Burning	Absorbable oxidized regenerated cellulose sheet	White Japanese Rabbit Sciatic nerve	- Electrophysiological evaluation (6 weeks) - Gross evaluation (qualitative evaluation—observation of Fontana’s bands) (6 weeks) - Histology on nerve and surrounding tissue (Masson’s trichrome) (6 weeks)	No significant differences between groups in electrophysiological evaluation. High adhesion between nerve and surrounding tissue in the damage group.
Ip et al., 2000 [15]	Section + suture	Early mobilization	Albino Rabbit Peroneal nerve	- Biomechanical examination (stretch test and peel test) (3 weeks)	No difference in the biomechanical features of the adhesions
